# Supplementary material for: DMRforPairs: identifying Differentially Methylated Regions between unique samples using array based methylation profiles
Source: BMC Bioinformatics. 2014 May 15;15:141. doi: 10.1186/1471-2105-15-141 (PMC4046028; doi:10.1186/1471-2105-15-141)
Supplement: Additional file 2 — DMRforPairs output for the comparison of A431-MCF7 and NA17018-NA17105. Please start from the HTML files in each folder. Available via the BMC Bioinformatics website. [file 1471-2105-15-141-S2.zip › 1394847754114233_MOESM2_ESM/A431_MCF7/figures/22756.pdf]

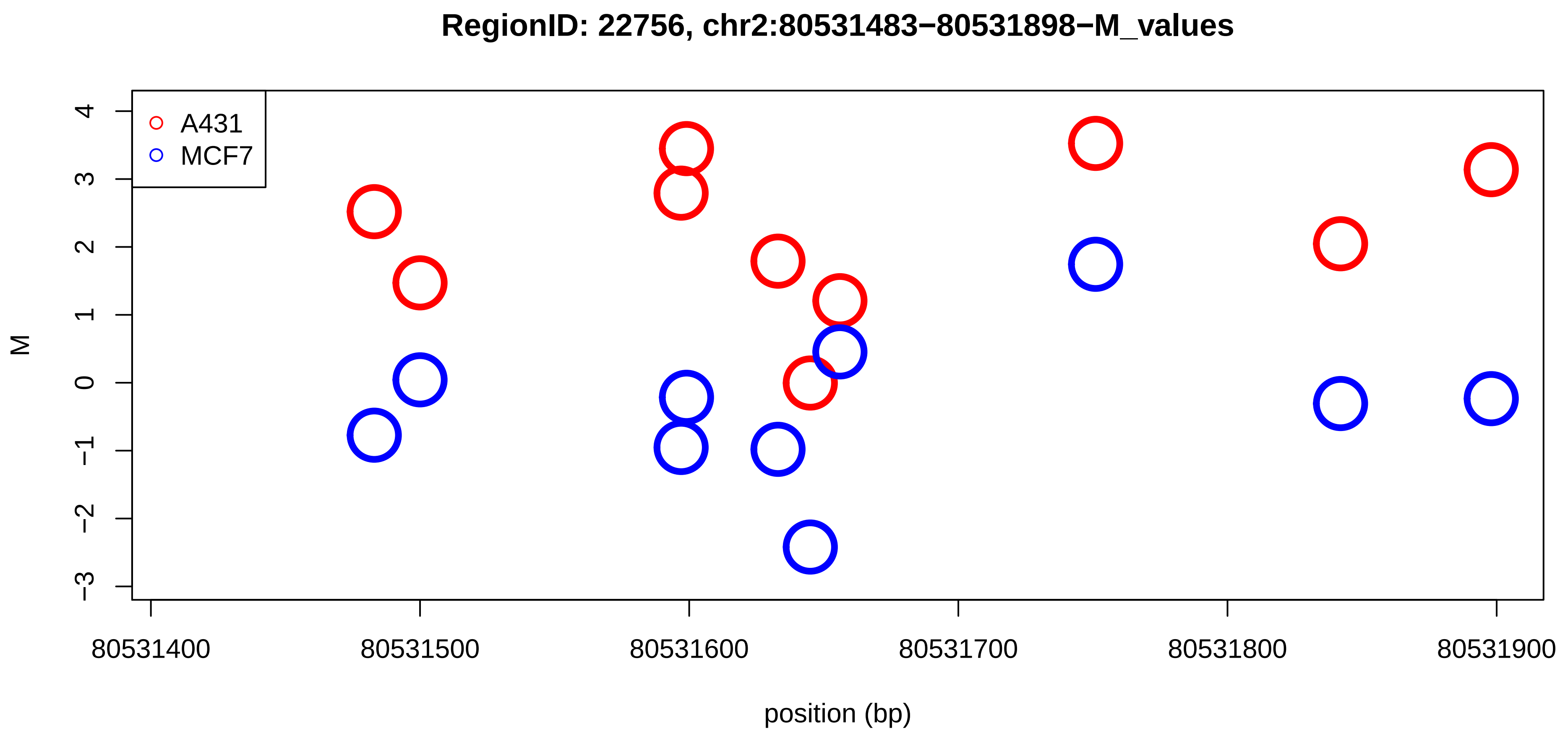

RegionID: 22756, chr2:80531483−80531898−M_values
A431
MCF7
M
4
3
2
1
0
−1
−2
−3
80531400
80531500
80531600
80531700
80531800
80531900
position (bp)

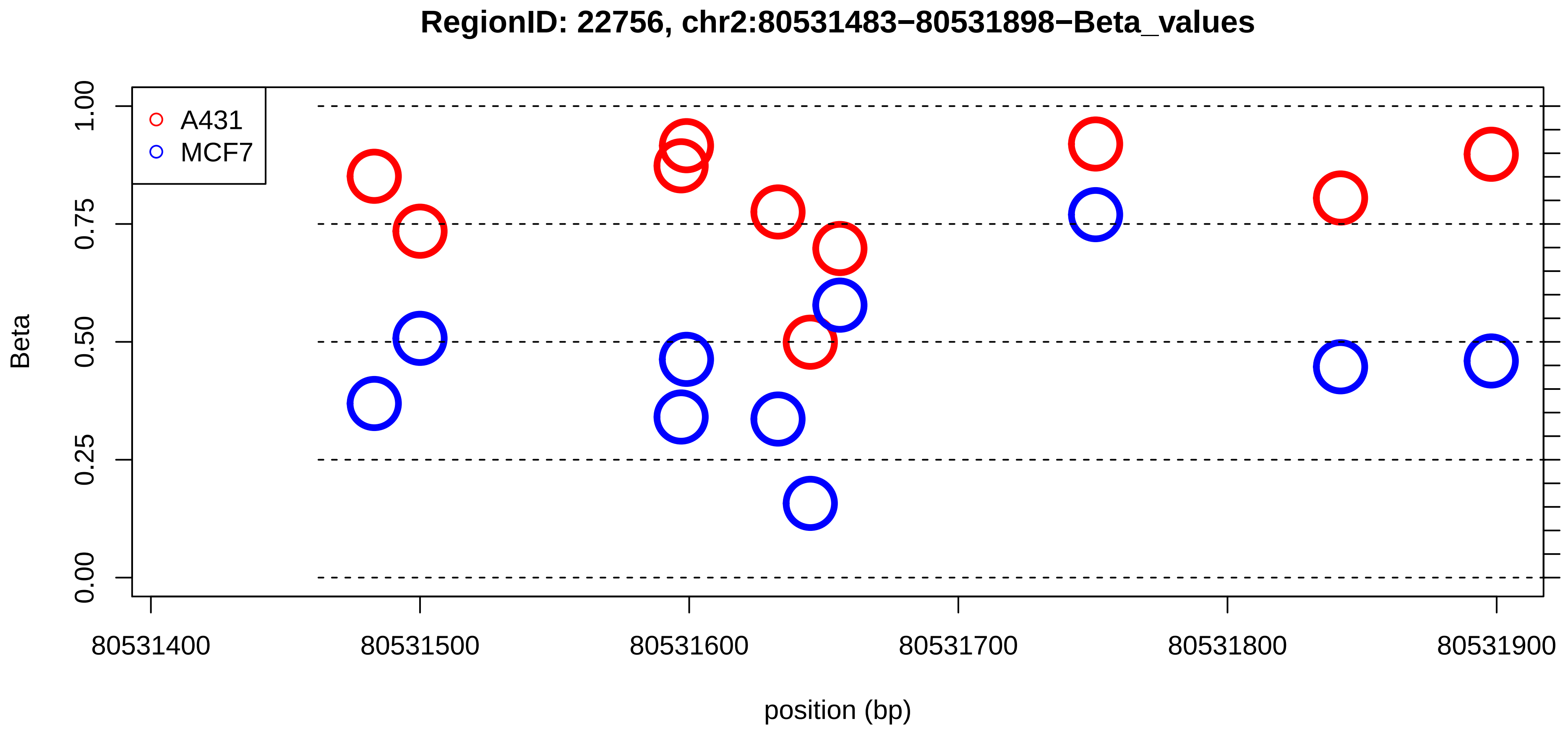

RegionID: 22756, chr2:80531483−80531898−Beta_values
A431
MCF7
Beta
1.00
0.75
0.50
0.25
0.00
80531400
80531500
80531600
80531700
80531800
80531900
position (bp)

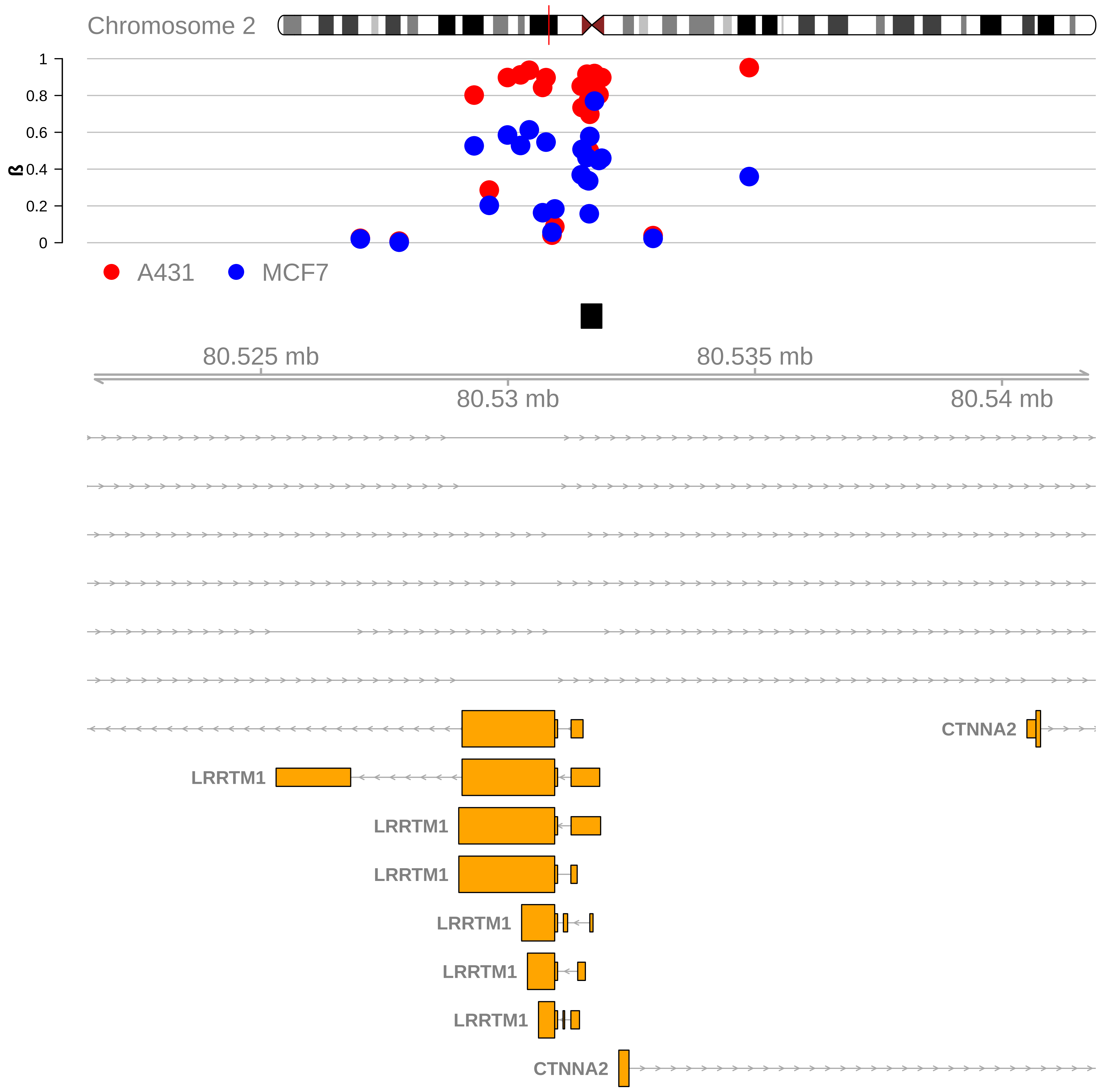

Chromosome 2
1
0.8
0.6
ß 0.4
0.2
0
A431
MCF7
80.525 mb
80.535 mb
80.53 mb
80.54 mb
CTNNA2
LRRTM1
LRRTM1
LRRTM1
LRRTM1
LRRTM1
LRRTM1
CTNNA2
